# Risk factors for HR-HPV persistence and residual or recurrent high-grade squamous intraepithelial neoplasia after LEEP

**DOI:** 10.3389/fmed.2026.1897304

**Published:** 2026-09-24

**Authors:** Liya Wang, Yiran Zhang, Yi Chen, Ying Xu, Lei Bao, Dan Cao, Dan Wu

**Affiliations:** 1 The Center for Cervical Disease, The International Peace Maternity and Child Health Hospital, School of Medicine, Shanghai Jiao Tong University, Shanghai, China; 2 Shanghai Key Laboratory of Embryo Original Disease, Shanghai, China

**Keywords:** cervical intraepithelial neoplasia, high-grade squamous intraepithelial lesions, human papillomavirus species, loop electrosurgical excision procedure, risk factors

## Abstract

**Background:**

To investigate factors associated with persistent high-risk human papillomavirus (HR-HPV) infection and residual or recurrent disease in patients with high-grade squamous intraepithelial lesions (HSIL) following loop electrosurgical excision procedure (LEEP).

**Methods:**

A total of 903 patients with pathologically confirmed HSIL who underwent LEEP between January 2021 and May 2024 were retrospectively enrolled. HR-HPV genotyping and cervical cytology were performed before and after surgery, and residual or recurrent HSIL was assessed by colposcopy during follow-up. Generalized estimating equation (GEE) logistic regression accounted for within-patient clustering of genotype-specific HPV observations. Multivariable logistic regression identified factors associated with HR-HPV persistence, and Firth's penalized logistic regression evaluated factors associated with residual or recurrent HSIL.

**Results:**

Persistent HR-HPV infection rates were 23.3%, 19.0%, and 17.5% at 6, 12, and 18 months after LEEP, respectively. GEE analysis showed significant differences in type-specific persistence among the *α*5/6, *α*7, and *α*9 species (*P* = 0.024), with higher persistence in *α*5/6 than *α*7 (27.1% vs. 14.0%; OR = 2.30, 95% CI, 1.09–4.85; adjusted *P* = 0.024), but no significant differences between *α*5/6 and *α*9 or between *α*7 and *α*9. Multiple HR-HPV infections (OR = 2.76, 95% CI, 1.96–3.89; *P* < 0.001) and menopausal status (OR = 2.06, 95% CI, 1.18–3.66; *P* = 0.011), and positive endocervical margins (OR = 2.54, 95% CI, 1.48–4.32; *P* = 0.007) were associated with HR-HPV persistence. During follow-up, 32 patients (3.5%) developed residual or recurrent HSIL (21 residual, 11 recurrent). Firth's penalized regression identified persistent type-specific HR-HPV infection (OR = 21.88, 95% CI, 8.25–72.76; *P* < 0.001), number of involved cervical quadrants (*P* = 0.025), endocervical involvement (*P* = 0.006), and surgical margin status (*P* < 0.001) as independently associated with residual or recurrent HSIL.

**Conclusions:**

*α*5/6 species showed a higher type-specific HR-HPV persistence than *α*7 but not *α*9 following LEEP. Multiple HR-HPV infections, menopausal status, and positive endocervical margins were associated with HR-HPV persistence. Persistent type-specific HR-HPV infection, multiple involved cervical quadrants, endocervical involvement, and positive surgical margins were independent predictors of residual or recurrent HSIL. Multicenter studies are warranted to validate integrating margin status with HPV genotyping for postoperative risk stratification.

## 1 Introduction

Cervical intraepithelial neoplasia (CIN) is a precancerous condition caused by persistent infection with high-risk human papillomavirus (HR-HPV). Without timely intervention, high-grade squamous intraepithelial lesions (HSIL) carry a substantially increased risk of progression to invasive cervical cancer (1). The loop electrosurgical excision procedure (LEEP) is widely used to remove dysplastic cervical tissue and effectively prevent progression to invasive disease. However, although LEEP eliminates the visible lesion, it does not necessarily eradicate the underlying viral infection (2, 3). Clinical follow-up studies have shown that approximately 5%–25% of patients develop residual or recurrent disease after LEEP, with persistent postoperative HR-HPV infection identified as the strongest predictor of recurrence (4, 5). In particular, patients with type-specific persistent HR-HPV infection may have up to a 30-fold higher risk of disease recurrence (6). Therefore, accurately identifying patients at high risk for persistent HR-HPV infection after surgery is of considerable clinical importance for optimizing individualized postoperative surveillance and improving the effectiveness of secondary cervical cancer prevention.

Previous studies have explored factors associated with postoperative HR-HPV persistence and residual or recurrent CIN. Potential risk factors reported include patient age, HR-HPV genotype, viral load, surgical margin status, higher pathological grade, endocervical glandular involvement, and host immune status (7–10). HR-HPV comprises a diverse group of genotypes that can be categorized into phylogenetic species groups, such as *α*5/6, *α*7, and *α*9, based on evolutionary relationships and genetic similarity (11). These phylogenetic groups differ substantially in their biological characteristics, including oncogenic potential and mechanisms of immune evasion (11, 12). Such genotype-specific differences may influence postoperative viral clearance and, consequently, clinical outcomes. However, existing clinical evidence remains limited, particularly with respect to large-scale studies evaluating HR-HPV persistence patterns in the Chinese population.

Therefore, this study aimed to investigate the characteristics of HR-HPV persistence and to identify independent associated factors for residual or recurrent HSIL after LEEP. The findings are intended to provide an evidence-based foundation for risk stratification and to support the development of more precise postoperative management strategies for patients with HSIL in the Chinese population.

## 2 Methods

### 2.1 Study population and inclusion criteria

A total of 903 patients with histologically confirmed HSIL (e.g., cervical CIN2, CIN3, and carcinoma *in situ*) who underwent LEEP at the Department of Cervical Pathology, International Peace Maternity and Child Health Hospital, between January 2021 and May 2024 were retrospectively enrolled. Inclusion criteria (1): Availability of HR-HPV genotyping results both before and after LEEP (2); Postoperative follow-up of at least 18 months with a minimum of two completed follow-up visits; (3) Complete clinical and follow-up data. Exclusion criteria: (1) Incomplete clinical data or loss to follow-up; (2) Receipt of adjuvant treatment for HSIL, such as hysterectomy or photodynamic therapy; (3) Prior cervical treatment, current pregnancy, or postpartum status within 8 weeks; (4) History of cervical cancer or other malignant tumors of the female genital tract; (5) History of pelvic radiotherapy or systemic chemotherapy; (6) Presence of autoimmune or systemic diseases or use of immunosuppressive medications. This study was approved by the Medical Research Ethics Committee of the International Peace Maternity and Child Health Hospital, affiliated with Shanghai Jiao Tong University (No. GKLW-A-2025-120-01).

### 2.2 Specimen collection and HPV genotyping

HR-HPV genotyping was performed using the Sansure® Human Papillomavirus DNA Diagnostic Kit (PCR Fluorescence Probing) (Sansure Biotech Inc.), which detects 15 high-risk HPV genotypes (16, 18, 31, 33, 35, 39, 45, 51, 52, 53, 56, 58, 59, 66, and 68). This assay has previously demonstrated high reproducibility and analytical reliability (13). Based on phylogenetic classification and genetic relatedness of papillomaviruses (12), HR-HPV genotypes in this study were categorized into three species groups: *α*5/6 (HPV types 51, 53, 56, and 66), *α*7 (HPV types 18, 39, 45, 59, and 68), and *α*9 (HPV types 16, 31, 33, 35, 52, and 58). Cervical cytology results were reported according to the Bethesda System and categorized as: no intraepithelial lesion or malignancy (NILM), atypical squamous cells of undetermined significance (ASC-US), atypical glandular cells (AGC), low-grade squamous intraepithelial lesion (LSIL), atypical squamous cells, cannot exclude HSIL (ASC-H), and HSIL.

### 2.3 Colposcopic examination

Colposcopic examination was performed when confirmatory test results were abnormal. Suspicious cervical lesions were biopsied based on colposcopic findings after application of acetic acid and Lugol's iodine staining. All colposcopic examinations were performed by eight experienced colposcopists at our center.

### 2.4 Surgical procedure

After obtaining informed consent, patients underwent cervical LEEP 3–7 days after the end of menstruation. The cervix was exposed with a vaginal speculum, and the lesion margins were delineated with Lugol's iodine solution. LEEP was performed using a VIO® 300 S electrosurgical system (Erbe Elektromedizin GmbH, Tübingen, Germany) in AutoCut mode at an output power of 40–50 W. Loop electrodes (10, 15, or 20 mm in diameter) or triangular electrodes (10 × 15 or 15 × 20 mm) of appropriate size were selected based on the lesion extent and cervical transformation zone (CTZ) type. The electrode was placed 3–5 mm beyond the visible lesion margin, and the entire CTZ together with the lesion was excised *en bloc* using a single, smooth, continuous pass. Piecemeal excision was performed when the lesion was too extensive to permit complete en-bloc resection.

For type I or II CTZs, the cone height was maintained at 10–15 mm, with a glandular excision depth of ≥5 mm. For patients with type III CTZ, lesions extending into the endocervical canal, or postmenopausal patients, a top-hat endocervical resection was performed after the primary ectocervical excision. A smaller-diameter loop electrode was used to remove suspicious or potentially involved deep endocervical tissue. This separate endocervical specimen was distinctly labeled and submitted for independent histopathological assessment.

No cauterization was applied to the margins of the ex-vivo surgical specimen, and routine extensive coagulation of the entire wound surface was avoided. All hemostatic procedures were limited to the in-situ cervical crater. Focal spot coagulation for sites of active bleeding was performed using a 5 mm monopolar ball-tip electrode at an output power of 30–40 W. All surgical procedures were performed by experienced gynecological specialists.

### 2.5 Follow-up protocol and evaluation criteria

Cervical cytology was performed at 3 months after LEEP, whereas HR-HPV genotyping and cervical cytology cotesting were performed at 6 months after LEEP. Thereafter, follow-up evaluations were performed every 6 months, with a minimum follow-up duration of 18 months. Follow-up assessments included cervical cytology, HR-HPV genotyping, and colposcopy with biopsy when clinically indicated. Type-specific persistent HR-HPV infection was defined as the detection of at least one identical HPV genotype at both the preoperative assessment and the 6-month postoperative HPV assessment, indicating persistence of the same genotype across the two consecutive HPV assessments. HPV clearance was defined as the complete absence of all HPV genotypes detected preoperatively at the 6-month postoperative HPV assessment. For the analysis of type-specific HR-HPV persistence, each detected HPV genotype was considered an observation, while within-patient correlation among multiple genotype observations was accounted for using GEE models. Postoperative residual disease was defined as biopsy-confirmed HSIL detected within 6 months after cervical conization. Postoperative recurrence was defined as the reappearance of pathologically confirmed HSIL more than 6 months after surgery, following initially negative cytology and HPV test results or normal biopsy findings within the first 6 months after the procedure.

### 2.6 Statistical analysis

Statistical analyses were performed using RStudio Desktop (Posit Software, PBC, Boston, MA, USA). Continuous variables were presented as the median with interquartile range [P50 (P25, P75)], while categorical variables were expressed as frequencies and percentages [n (%)]. The Kolmogorov–Smirnov test was used to assess normality; non-normally distributed continuous variables were compared using the rank-sum test, and categorical variables using the chi-square test.

Because individual patients could harbor multiple HPV genotypes, genotype-specific HPV observations within the same patient were correlated. Generalized estimating equation (GEE) logistic regression models with a logit link were therefore used, with the outpatient registration number as the clustering variable. GEE models were used to compare multiple infection rates and type-specific HR-HPV persistence rates across HPV phylogenetic species. Results were expressed as estimated rates with 95% confidence intervals (CIs), and pairwise comparisons were performed with Bonferroni adjustment for multiple comparisons. Variables with a *P* value < 0.05 in univariable analyses were included in a multivariable logistic regression model to identify independent associated factors for persistent HR-HPV infection. For the analysis of factors associated with residual or recurrent HSIL, candidate variables were initially selected from univariable analyses using a *P* value < 0.05. Potential collinearity among candidate variables was assessed using Cramer's V. Given the limited number of outcome events (32 cases) and the sparse categorical data, Firth's penalized logistic regression was used to reduce small-sample bias and mitigate separation. Backward elimination was used to obtain the final model. Results were reported as odds ratios (ORs) with corresponding 95% CIs.

All statistical tests were two-sided, and a *P* value < 0.05 was considered statistically significant unless otherwise specified for multiplicity-adjusted comparisons.

## 3 Results

### 3.1 Patient characteristics

A total of 903 patients were included in this study. The baseline clinical characteristics of the cohort are summarized in Table 1. The median age was 41 years, with an interquartile range (IQR, 25th-75th percentile) of 35–50 years. Most patients were premenopausal (688/903, 76.2%). Abnormal cervical cytology was observed in 556 patients (61.6%). Postoperative pathological examination revealed cervical intraepithelial neoplasia grade 2 (CIN2) in 464 patients (51.4%), CIN3 in 435 patients (48.2%), and carcinoma *in situ* (CIS) in 4 patients (0.4%). Regarding HR-HPV infection status, 623 patients (69.0%) had single-type HR-HPV infection, 206 (22.8%) had infection with two HR-HPV genotypes, and 74 (8.2%) had infection with three or more genotypes. The HR-HPV persistence rates at 6, 12, and 18 months after surgery were 23.3% (210/903), 19.0% (172/903), and 17.5% (158/903), respectively. During the 18-month postoperative follow-up period, residual or recurrent HSIL was identified in 32 patients, corresponding to an overall rate of 3.5%.

**Table 1:** Characteristics of the study population (*n* = 903).

Characteristics	Parameters	Case (%)
Age (years)		
	Median	41
	25th-75th percentile	35–50
Menopausal		
	Postmenopausal	215 (23.8)
	Premenopausal	688 (76.2)
Number of pregnancies		
	<3	546 (60.5)
	≥3	357 (39.5)
Parity		
	＜2	674 (74.6)
	≥2	229 (25.4)
Preoperative Cervical cytology^a^		
	NILM	347 (38.4)
	ASCUS/AGC	213 (23.6)
	LSIL	148 (16.4)
	ASC-H	87 (9.6)
	HSIL	108 (12.0)
Colposcopic impression of high-grade lesion		
	Yes	838 (92.8)
	No	65 (7.2)
Colposcopic pathology (without ECC)		
	Chronic cervicitis	12 (1.3)
	CIN1	130 (14.4)
	CIN2	395 (43.7)
	CIN3	366 (40.5)
Cervical transformation zone (CTZ)		
	I	369 (40.9)
	II	246 (27.2)
	III	288 (31.9)
Endocervical involvement		
	Negative	558 (61.8)
	Positive (CIN1)	112 (12.4)
	Positive (CIN2-3)	233 (25.8)
Glandular involvement		
	Yes	549 (60.8)
	No	354 (39.2)
Postoperative pathology		
	CIN2	464 (51.4)
	CIN3	435 (48.2)
	CIS	4 (0.4)
Surgical margin		
	Negative	765 (84.7)
	Ectocervical margin	43 (4.8)
	Endocervical margin	77 (8.5)
	Basal margin	18 (2.0)
Postoperative HR-HPV persistence		
	6 months	210 (23.3)
	12 months	172 (19.0)
	18 months	158 (17.5)
Residual or recurrent HSIL		
	Yes	32 (3.5)
	No	871 (96.5)

Values are presented as median (25th-75th percentile) for continuous variables (age) or *n* (%) for categorical variables. ^a^Preoperative cervical cytology results (≤3 months before LEEP).

HR-HPV, high-risk human papillomavirus; NILM, no intraepithelial lesion or malignancy; ASC-US, atypical squamous cells of undetermined significance; AGC, atypical glandular cells; LSIL, low-grade squamous intraepithelial lesion; ASC-H, atypical squamous cells, cannot exclude HSIL; HSIL, high-grade squamous intraepithelial lesion; CIN, cervical intraepithelial neoplasia; CIS, carcinoma *in situ*.

Among the 903 patients, 347 had preoperative cervical cytology reported as NILM. Of these patients, 296 had HSIL on preoperative biopsy and therefore met standard indications for LEEP. The remaining 51 patients showed CIN1 on preoperative biopsy and underwent diagnostic LEEP based on individual clinical assessment. Of these 51 patients, 20 had a type III CTZ, in which the squamocolumnar junction was not fully visible, limiting the reliability of colposcopic assessment and raising concern that high-grade lesions might have been missed because of sampling limitations. The remaining 31 patients had CIN1 on biopsy but exhibited clinical features associated with an increased risk of underlying high-grade disease, including HPV16/18 positivity or colposcopic findings highly suggestive of HSIL. After comprehensive counseling regarding the potential benefits and risks of surveillance versus diagnostic excision, and following exclusion of contraindications to the procedure, these patients underwent diagnostic LEEP through a shared decision-making process. Postoperative histopathological examination demonstrated HSIL in all 51 patients, indicating that the preoperative biopsies had underestimated the severity of the underlying lesions.

### 3.2 Persistence and multiple infection rates of different HPV genotypes

The 903 patients were categorized into three groups according to HPV phylogenetic species: *α*5/6, *α*7, and *α*9. The distributions of preoperative multiple HR-HPV infection and postoperative type-specific HR-HPV persistence across the three species groups are presented in Supplementary Tables S1. Among all detected genotype-specific HPV observations, *α*9 was the predominant phylogenetic species. HPV16 was the most frequently detected genotype (373 cases, 41.3%), followed by HPV52 (247 cases, 27.4%) and HPV58 (159 cases, 17.6%). In contrast, genotypes belonging to the *α*7 and *α*5/6 species were detected less frequently.

After accounting for within-patient correlation using GEE logistic regression, the estimated rates of multiple HR-HPV infection were 44.1% (95% CI, 40.4–48.0%) in the *α*9 group, 71.4% (95% CI, 63.9–77.9%) in the *α*7 group, and 74.7% (95% CI, 67.1–81.0%) in the *α*5/6 group. A significant overall difference was observed among the three species groups (*P* < 0.001). Pairwise comparisons showed that the multiple infection rate was significantly lower in the *α*9 group than in both the *α*7 and *α*5/6 groups (both adjusted *P* < 0.001), whereas no significant difference was observed between the *α*7 and *α*5/6 groups (adjusted *P* = 1.000) (Table 2 and Supplementary Table S2).

**Table 2:** GEE-adjusted comparison of multiple infection rates by HPV genospecies.

Species	Genotype observations	Patient clusters	Multiple infection observations	GEE estimated multiple infection (95% CI)	Overall *P* value
*α*9	954	797	421	44.1% (40.4%–48.0%)	<0.001
α7	168	153	120	71.4% (63.9%–77.9%)	
α5/6	166	143	124	74.7% (67.1%–81.0%)	

For postoperative species-specific HR-HPV persistence, the GEE-estimated marginal probabilities of persistence were 18.8% (95% CI, 16.3–21.6%) for *α*9, 14.0% (95% CI, 9.3–20.3%) for *α*7, and 27.1% (95% CI, 20.1–35.5%) for *α*5/6. The overall difference among the three species groups was statistically significant (*P* = 0.024). In pairwise comparisons with Bonferroni adjustment, the persistence rate was significantly higher in the *α*5/6 group than in the *α*7 group (OR = 2.30, 95% CI, 1.09–4.85, adjusted *P* = 0.024). However, the differences between *α*5/6 and *α*9 (OR = 1.61, 95% CI, 0.95–2.71, adjusted *P* = 0.091) and between *α*7 and *α*9 (OR = 0.70, 95% CI, 0.40–1.24, adjusted *P* = 0.403) were not statistically significant (Table 3 and Supplementary Table S3). Among individual HPV genotypes, HPV18 showed the lowest persistence rate, at 4.4% (3/68).

**Table 3:** GEE-adjusted comparison of type-specific HR-HPV persistence by HPV genospecies.

Species	Genotype observations	Patient clusters	Same-type persistence	GEE estimated persistence (95% CI)	Overall *P* value
α9	954	797	186	18.8% (16.3%–21.6%)	0.024
α7	168	153	29	14.0% (9.3%–20.3%)	0.024
α5/6	166	143	56	27.1% (20.1%–35.5%)	0.024

### 3.3 Factors associated with HR-HPV persistence after LEEP

A total of 210 patients (23.3%) demonstrated HR-HPV persistence at 6 months after LEEP. The baseline characteristics of patients with and without persistent infection are summarized in Table 4. The median age of the persistent infection group was significantly higher than that of the non-persistent group [46.0 (35.2–58.0) vs. 40.0 (35.0–47.0) years, *P* < 0.001]. Univariate analysis identified age, menopausal status, multiple HR-HPV infections, cervical transformation zone (CTZ) type, endocervical canal involvement, and surgical margin status as factors significantly associated with HR-HPV persistence (*P* < 0.05). In contrast, gravidity and parity, cervical cytology results, number of involved cervical quadrants, glandular involvement, colposcopic biopsy pathology and postoperative pathological grade were not significantly associated with HR-HPV persistence (*P* > 0.05).

**Table 4:** Risk factors associated with persistent HR-HPV infection after surgical treatment.

Characteristics		Postop HR-HPV persistence (until 6 mon)		
		Negative (*n* = 693)	Positive (*n* = 210)	*P*-value
Age (years)		40.0 [35.0, 47.0]	46.0 [35.2, 58.0]	<0.001
Age (years)	≤30	71 (77.2%)	21 (22.8%)	<0.001
	31–45	412 (83.4%)	82 (16.6%)	
	≥46	210 (66.2%)	107 (33.8%)	
Menopausal	Postmenopausal	129 (60.0%)	86 (40.0%)	<0.001
	Premenopausal	564 (82.0%)	124 (18.0%)	
Number of pregnancies	<3	424 (77.7%)	122 (22.3%)	0.471
	≥3	269 (75.4%)	88 (24.6%)	
Parity	<2	520 (77.2%)	154 (22.8%)	0.685
	≥2	173 (75.5%)	56 (24.5%)	
Preoperative HPV type	Single	516 (82.8%)	107 (17.2%)	<0.001
	Multiple	177 (63.2%)	103 (36.8%)	
Cervical cytology	NILM	274 (79.0%)	73 (21.0%)	0.138
	ASCUS/AGC	164 (77.0%)	49 (23.0%)	
	LSIL	102 (68.9%)	46 (31.1%)	
	ASC-H	66 (75.9%)	21 (24.1%)	
	HSIL	87 (80.6%)	21 (19.4%)	
Cervical transformation zone (CTZ)	I	305 (82.7%)	64 (17.3%)	<0.001
	II	199 (80.9%)	47 (19.1%)	
	III	189 (65.6%)	99 (34.4%)	
Number of cervical quadrants involved	0	82 (68.9%)	37 (31.1%)	0.178
	1	447 (77.5%)	130 (22.5%)	
	2	112 (77.2%)	33 (22.8%)	
	3	26 (86.7%)	4 (13.3%)	
	4	26 (81.2%)	6 (18.8%)	
Endocervical involvement	Negative	443 (79.4%)	115 (20.6%)	0.024
	Positive (CIN1)	86 (76.8%)	26 (23.2%)	
	Positive (CIN2-3)	164 (70.4%)	69 (29.6%)	
Glandular involvement	Yes	431 (78.5%)	118 (21.5%)	0.139
	No	262 (74.0%)	92 (26.0%)	
Colposcopic pathology	Chronic cervicitis	10 (76.9%)	3 (23.1%)	0.223
	CIN1	92 (71.3%)	37 (28.7%)	
	CIN2	333 (75.9%)	106 (24.1%)	
	CIN3	258 (80.1%)	64 (19.9%)	
Postoperative pathology	CIN2	343 (73.9%)	121 (26.1%)	0.116
	CIN3	347 (79.8%)	88 (20.2%)	
	CIS/AIS	3 (75.0%)	1 (25.0%)	
Surgical margin	Negative	603 (78.8%)	162 (21.2%)	0.001
	Ectocervical margin	32 (74.4%)	11 (25.6%)	
	Endocervical margin	46 (59.7%)	31 (40.3%)	
	Basal margin	12 (66.7%)	6 (33.3%)	

Variables with *P* < 0.05 in the univariate analysis were included in the multivariate logistic regression model (Table 5). The results showed that multiple HR-HPV infections (OR = 2.76, 95% CI, 1.96–3.89, *P* < 0.001) and menopausal status (OR = 2.06, 95% CI, 1.18–3.66, *P* = 0.011) were independent associated factors for postoperative HR-HPV persistence. Surgical margin status was significantly associated with postoperative HR-HPV persistence (overall *P* = 0.007). Compared with a negative margin, a positive endocervical margin was associated with higher odds of persistence (OR = 2.54, 95% CI, 1.48–4.32). In contrast, age, CTZ type, and endocervical canal involvement were not independently associated with HR-HPV persistence in the final model.

**Table 5:** Multivariate logistic regression analysis of factors associated with persistent HR-HPV infection after surgical treatment.

Characteristics	Parameters	OR	95% CI	*P*-value
Age (years)	≤30			0.386
	31–45	0.67	0.38, 1.20	
	≥46	0.76	0.37, 1.56	
Menopausal	Premenopausal			0.011
	Postmenopausal	2.06	1.18, 3.66	
Preoperative HPV type	Single			<0.001
	Multiple	2.76	1.96, 3.89	
Cervical transformation zone (CTZ)	I			0.145
	II	1.06	0.68, 1.65	
	III	1.55	0.97, 2.45	
Endocervical involvement	Positive (CIN2-3)			0.302
	Negative	0.76	0.51, 1.12	
	Positive (CIN1)	0.7	0.40, 1.22	
Surgical margin	Negative			0.007
	Ectocervical margin	1.39	0.64, 2.84	
	Endocervical margin	2.54	1.48, 4.32	
	Basal margin	1.67	0.54, 4.67	

### 3.4 Factors associated with HSIL residual or recurrence after LEEP

During follow-up, 32 patients (3.5%) developed residual or recurrent HSIL. Factors associated with residual or recurrent HSIL are presented in Supplementary Table S4. The median age of patients in the residual or recurrent group was 52.0 years (IQR, 42.0–59.0), which was significantly higher than that of patients without residual or recurrent disease [40.0 years (IQR, 34.5–49.0); *P* < 0.001]. In univariable analyses, menopausal status, number of pregnancies, persistent HR-HPV infection, cervical cytology, number of involved cervical quadrants, endocervical involvement, and surgical margin status were significantly associated with HSIL residual or recurrence (all *P* < 0.05). In contrast, parity, multiple HPV infection, CTZ type, glandular involvement, colposcopic biopsy pathology, and postoperative pathological grade were not significantly associated with the outcome (all *P* > 0.05).

Given the small number of outcome events (32 cases) and the sparse distribution of some categorical variables, we used Firth's penalized logistic regression for the multivariable analysis to address potential small-sample bias and separation. Variables with *P* < 0.05 in the univariable analyses were considered for the multivariable model. Collinearity was assessed using Cramer's V. Age category was excluded because of its strong association with menopausal status. The other candidate variables were entered into the model, and backward elimination was used to remove variables that did not contribute significantly to the model. The final model included persistent type-specific HR-HPV infection, number of involved cervical quadrants, endocervical lesion involvement, and surgical margin status (Table 6).

**Table 6:** Firth's penalized logistic regression analysis of factors associated with residual or recurrent HSIL after LEEP.

Characteristics	Parameters	OR	95% CI	*P*-value
Persistent HPV infection	No			<0.001
	Yes	21.88	8.25,72.76	
Number of cervical quadrants involved	0			0.025
	1	2.39	0.66,10.82	
	2	7.22	1.86, 34.43	
	3	2.95	0.02, 39.15	
	4	11.93	1.39, 103.06	
Endocervical involvement	Negative			0.006
	Positive (CIN1)	2.33	0.51, 9.21	
	Positive (CIN2-3)	4.95	1.87, 13.90	
Surgical margin	Negative			<0.001
	Ectocervical margin	9.85	2.44, 36.30	
	Endocervical margin	5.88	2.18, 15.90	
	Basal margin	40.6	8.86, 188.88	

Persistent type-specific HR-HPV infection showed a strong association with HSIL residual or recurrence (OR = 21.88, 95% CI,8.25–72.76, *P* < 0.001). The number of involved cervical quadrants was also associated with the outcome (*P* = 0.025), with higher odds for involvement of two or four quadrants than for no quadrant involvement. Endocervical involvement remained significantly associated with HSIL residual or recurrence (*P* = 0.006); in particular, CIN2-3 involvement was associated with higher odds than no endocervical involvement (OR = 4.95, 95% CI,1.87–13.90). Surgical margin status showed a similarly strong association (*P* < 0.001). The largest estimated effect was observed for basal margin involvement (OR = 40.60, 95% CI,8.86–188.88).

Among the 32 patients who developed residual or recurrent HSIL during follow-up, 21 (65.6%) were classified as having residual disease and 11 (34.4%) as having recurrent disease. An exploratory comparison of clinical and pathological characteristics between the two groups is presented in Table 7. Persistent HPV infection was detected in 18 of 21 patients (85.7%) with residual disease and 10 of 11 patients (90.9%) with recurrent disease (*P* = 1.000). Positive surgical margins were observed in 17 patients (81.0%) with residual disease and 5 patients (45.5%) with recurrent disease (*P* = 0.056). Although surgical margin status showed a trend toward a difference between the two groups, neither persistent HPV infection nor surgical margin status differed significantly between patients with residual and recurrent disease.

**Table 7:** Exploratory comparison of clinical characteristics of residual or recurrent HSIL.

Characteristics	Parameters	Recurrent (*n* = 11)	Residual (*n* = 21)	*P*-value
Persistent HPV infection	Yes	10 (90.9)	18 (85.7%)	1.000
	No	1 (9.1%)	3 (14.3%)	
Surgical margin	Negative	6 (54.5%)	4 (19.0%)	0.056
	Positive	5 (45.5%)	17 (81.0%)	

A total of 57 genotype-specific HPV observations were identified preoperatively in the 32 patients. Of these, 42 (73.7%) belonged to the *α*9 species, 10 (17.5%) to *α*5/6, and 5 (8.8%) to *α*7 (Supplementary Table S5). In the overall study cohort, 1,288 genotype-specific HPV observations were identified, of which 954 (74.1%) belonged to *α*9, 168 (13.0%) to *α*7, and 166 (12.9%) to *α*5/6 (Table 2). Thus, the predominance of *α*9 among patients with residual or recurrent HSIL was consistent with its high prevalence in the overall study population. These data describe the distribution of HPV genotypes among patients with residual or recurrent HSIL and do not imply that *α*9 was associated with the highest risk of residual or recurrent HSIL. Notably, 28 of 32 patients (87.5%) with residual or recurrent HSIL had persistent HR-HPV infection.

## 4 Discussion

This study examined the independent associated factors associated with HR-HPV persistence and residual or recurrent HSIL after LEEP. Our findings provide additional evidence to support risk-stratified postoperative management and contribute to optimizing strategies for the secondary prevention of cervical cancer.

### 4.1 Characteristics and clinical implications of HR-HPV persistence

In the present study, HR-HPV clearance rates at 6, 12, and 18 months after LEEP were 76.7%, 81.0%, and 82.5%, respectively. Although these rates were slightly lower than those reported in previous studies (3, 14), the overall temporal pattern of postoperative HPV clearance was generally consistent. The discrepancy may be attributable to differences in patient inclusion criteria, such as surgical margin status and treatment modalities, as well as variations in follow-up intervals across studies. Our findings indicate that more than two-thirds of patients achieved HR-HPV clearance within 6 months after surgery. Moreover, the first postoperative year appears to represent a critical period for viral clearance, with relatively few additional clearance events observed after 12 months. These results highlight the importance of close surveillance during the early postoperative period while also highlighting the necessity of continued long-term follow-up.

Previous studies have reported considerable variability in persistence rates among different HR-HPV genotypes (7, 15–17). Gosvig CF et al. found that HPV *α*5/6 species were more likely to persist than *α*9 species (18). In our analysis, *α*5/6 species showed the highest GEE-estimated type-specific persistence rate and a significantly higher persistence rate than *α*7, whereas the difference between *α*5/6 and *α*9 was not statistically significant. This discrepancy may reflect differences in study populations, HPV detection methods, follow-up timing, sample size, and statistical approaches. Importantly, our analysis accounted for within-patient clustering among multiple HPV genotype observations using GEE models, which may have affected the precision of the between-species comparisons. The higher numerical persistence rate observed for *α*5/6 in our study may represent a potential difference in persistence across phylogenetic groups, but the evidence was insufficient to conclude that *α*5/6 has a significantly higher persistence propensity than *α*9. In contrast, HPV18 exhibited the lowest persistence rate (4.4%) among all detected genotypes. These findings are consistent with previous reports (18) and may reflect inherent biological differences among HPV phylogenetic groups (19, 20). Although *α*5/6 species have relatively low prevalence in the general population, they may possess stronger immune evasion capabilities and enhanced adhesion to cervical epithelial cells, thereby facilitating postoperative viral persistence. Conversely, *α*7 species, particularly HPV18, may induce stronger host immune responses due to greater immunogenicity, resulting in more efficient viral clearance (21).

Furthermore, the multiple infection rate was significantly lower in the *α*9 group than in the *α*7 and *α*5/6 groups, which is consistent with earlier studies (16). Although *α*9 genotypes demonstrate moderate persistence and relatively low rates of multiple infection, their high prevalence and strong carcinogenic potential warrant continued prioritization during postoperative surveillance. In contrast, while *α*5/6 genotypes exhibit a higher likelihood of persistence, their oncogenic potential may be lower than that of *α*7 and *α*9 species, suggesting that some infections within this group may represent solely bystanders (18).

HPV53, which belongs to the *α*5/6 species, was fully genotyped in this study. It showed a high co-infection rate (81.8% pre-LEEP) and a postoperative persistence rate of 36.4%, which is consistent with the general behavior of this phylogenetic group. A previous study has suggested that HPV53 exhibits relatively weak oncogenic potential and is more commonly associated with mild low-grade cervical lesions and slow disease progression, and it is generally considered a genotype with limited independent contribution to HSIL development (22). The clinical significance of isolated persistent HPV53 infection requires further investigation.

### 4.2 Risk factors for postoperative HR-HPV persistence and residual or recurrence HSIL

In this study, 23.3% (210/903) of patients developed postoperative HR-HPV persistence. Multiple HR-HPV infection was identified as a significant independent risk factor for postoperative persistence, associated with 2.76-fold higher odds compared with single-type infection (OR = 2.76, 95% CI, 1.96–3.89). This finding is consistent with previous studies (8, 16, 23, 24). Multiple HR-HPV infections may increase the likelihood of immune evasion, impair host immune responses, and ultimately reduce viral clearance efficiency (25). The relationship between multiple HR-HPV infections and residual or recurrent disease remains controversial. Some studies have reported that multiple HR-HPV infections are associated with a modest but significant increase in the risk of postoperative HSIL residual or recurrence, suggesting that they may serve as a potential prognostic indicator of unfavorable outcomes after treatment for high-grade cervical lesions (16, 24). In contrast, other studies propose that multiple HR-HPV infections largely represent random co-infections, with each HPV type independently contributing to cervical lesion development through cumulative rather than synergistic effects (26). The latter interpretation is consistent with our findings, which indicate that multiple HPV infections were associated with HR-HPV persistence but not with HSIL residual or recurrence. These inconsistencies across studies may be attributable to differences in sample size, population characteristics, and follow-up duration, highlighting the need for further validation in larger prospective cohorts.

Previous studies and meta-analyses have confirmed that postoperative persistence of the same HR-HPV genotype is the strongest predictor of residual or recurrent HSIL, whereas newly acquired HR-HPV infections post-surgery carry almost no risk of recurrence (6, 20, 27). In our study, 87.5% (28/32) of residual or recurrent HSIL exhibited persistence of the same HR-HPV genotype before and after surgery. Firth's penalized logistic regression further identified postoperative type-specific HR-HPV persistence as an independent risk factor for residual or recurrent HSIL, corroborating previous findings (17, 28–31).

Positive surgical margins have been widely recognized as important risk factors for postoperative HPV persistence and lesion residual or recurrence (7, 9, 15, 18). However, relatively few studies have conducted detailed analyses distinguishing among endocervical, ectocervical, and basal margin involvement. In the present study, positive endocervical margins were identified as a strong independent predictor of postoperative HR-HPV persistence (OR = 2.54). Positive endocervical margins indicate possible presence of residual lesions deep within the endocervical canal, which may contribute to persistent HR-HPV infection.

Xiao et al. found that positive endocervical and basal surgical margins were strong predictors of residual disease (32). Meta-analyses (33, 34) confirmed that positive surgical margins significantly increase the risks of residual or recurrent HSIL after LEEP. However, margin pathology has low sensitivity for predicting recurrence, and sole reliance on margin status tends to miss high-risk patients. Accordingly, surgical margins are not recommended as an isolated prognostic biomarker, and postoperative risk stratification for HSIL guided by HR-HPV genotyping is preferred in clinical practice. A major limitation of these meta-analyses is the absence of complete HPV genotyping data in nearly all incorporated primary studies, which precluded differentiation between genotype-specific persistent HR-HPV infection and newly acquired HPV infection. Consequently, the confounding effect of type-specific HPV persistence on the prognostic value of margin status could not be precisely estimated. A recent large-scale cohort study (35) reported that surgical margin status remained an independent predictor of post-LEEP recurrence after adjustment for type-specific persistent HPV infection, although its predictive strength was substantially attenuated. The authors concluded that type-specific persistent HPV infection is the dominant independent predictor of recurrence, whereas margin positivity exhibited a graded association, supporting the complementary contributions of both incomplete excision-related residual disease and subsequent virologically driven neoplastic recurrence to treatment failure.

In our study, surgical margin status was also strongly associated with residual or recurrent HSIL, with basal margin positivity showing the highest odds compared with negative margins (OR = 40.60, 95% CI, 8.86–188.88). This contrasts with a previous study (35), in which the predictive effect of margin status was markedly attenuated upon HPV adjustment. However, the strong association observed for basal margin positivity should be interpreted cautiously. In the exploratory analysis, positive surgical margins were more common in patients with residual disease than in those with recurrent disease (81.0% vs. 45.5%), although this difference did not reach statistical significance (*P* = 0.056). In contrast, persistent HPV infection was highly prevalent in both groups (85.7% vs. 90.9%, *P* = 1.000). These findings suggest that margin status may be more closely related to residual disease, whereas persistent HPV infection may be relevant to both residual disease and later recurrence. However, the small number of recurrent cases (*n* = 11) limited the statistical power to distinguish these patterns. Therefore, residual and recurrent HSIL were combined in the primary multivariable analysis to preserve statistical power.

Although Firth's penalized logistic regression was used to reduce small-sample bias and address separation, some estimates remained imprecise, as reflected by the wide confidence intervals, particularly for categories with few events. Larger multicenter prospective studies are needed to determine whether margin status provides additional prognostic information beyond persistent HPV infection and to determine whether incorporating margin status into HPV-based postoperative risk stratification improves clinical decision-making.

The association between menopausal status and HPV persistence remains controversial. Although some previous studies have reported that menopausal status is not an independent risk factor for HPV persistence (36, 37), our study identified menopausal status as an independent predictor of postoperative persistence, with 2.06-fold higher odds compared with premenopausal women (OR = 2.06, 95% CI, 1.18–3.66). This finding is consistent with the majority of published reports (7, 15, 17, 23, 25, 38, 39). In postmenopausal women, declining estrogen levels lead to retraction of the cervical transformation zone into the endocervical canal and reduced local immune function. These changes not only increase the difficulty of complete lesion excision but may also impair the host immune system's ability to eliminate residual viral infection (25, 39). In our multivariate analysis, age did not retain independent significance after stratification, likely due to substantial strong association between age and menopausal status. Compared with chronological age, menopausal status more directly reflects the host's endocrine and immunological environment, which may explain why its effect overshadowed that of age in the final model.

In addition, our study showed that involvement of multiple cervical quadrants and the endocervical involvement were associated with residual or recurrent HSIL after LEEP. Compared with patients with no involved cervical quadrants, those with involvement of two or four cervical quadrants had higher odds of residual or recurrent HSIL (OR = 7.22 and 11.93, respectively). Endocervical involvement was also associated with increased odds of residual or recurrent HSIL (OR = 4.95, 95% CI, 1.87–13.90). These findings may reflect the difficulty of achieving complete excision when the disease is more extensive or extends into the endocervical canal, potentially increasing the likelihood of residual or recurrent HSIL after LEEP. These characteristics may therefore be useful in identifying patients who require closer postoperative surveillance. However, the relatively small number of outcome events and wide confidence intervals for some estimates warrant cautious interpretation, and these findings should be confirmed in larger prospective cohorts. Notably, 51 patients with preoperative NILM cytology and biopsy-confirmed CIN1 were ultimately upgraded to HSIL on postoperative LEEP specimens, highlighting the inherent sampling limitations of colposcopy-guided targeted biopsy. Normal cervical cytology cannot reliably rule out underlying HSIL, as false-negative results are clinically possible.

The 2019 ASCCP guidelines recommend treatment for histologic HSIL, whereas observation is generally preferred for isolated histologic CIN1. However, diagnostic excision may be acceptable in selected patients with high-grade cytologic abnormalities despite biopsy showing CIN1 or less, particularly when the squamocolumnar junction or the upper limit of the lesion is not fully visualized (40). In the present cohort, patients who underwent diagnostic LEEP for biopsy-proven CIN1 all presented with high-risk clinical features, including a type III CTZ, HPV16/18 positivity, or colposcopic changes highly suggestive of occult high-grade lesions. All excisions were individually determined after detailed patient counseling and shared decision-making. The uniform postoperative pathological upgrade in these cases further indicates that these clinical risk factors strongly suggest undiagnosed HSIL that may be missed by limited biopsy sampling. Even so, strict adherence to clinical indications remains critical to avoid overtreatment, and the potential reproductive risks of cervical excision should be carefully balanced before surgical intervention.

### 4.3 Limitations

This study has several limitations. First, as a single-center retrospective study, its generalizability may be limited, and potential selection bias cannot be completely excluded. Second, the follow-up duration may not have been sufficient to fully evaluate long-term recurrence risk after LEEP. Third, the limited number of outcome events (only 32 patients with residual or recurrent HSIL) and the sparse distribution of some categorical variables may have contributed to model instability and reduced the precision of some risk estimates, as reflected by the wide confidence intervals. In addition, because type-specific HPV persistence was defined using the 6-month postoperative HPV assessment, its temporal relationship with residual disease diagnosed before the 6-month assessment cannot be established. Future multicenter prospective studies with larger sample sizes are needed to validate the clinical utility of HPV-specific classification and margin status assessment in postoperative management. Such studies should also explore individualized follow-up strategies based on risk stratification and further investigate the interactions between different HR-HPV species and the host immune microenvironment. These efforts may provide stronger evidence for optimizing secondary prevention strategies for cervical cancer.

## 5 Conclusion

This study systematically evaluated the factors associated with HR-HPV persistence and HSIL residual or recurrence after LEEP. Preoperative multiple HR-HPV infection, menopausal status, and positive endocervical margins were associated with postoperative HPV persistence, while persistent type-specific HPV infection, involvement of multiple cervical quadrants, endocervical involvement, and surgical margin positivity were associated with residual or recurrent HSIL. Although *α*5/6 showed a higher persistence rate than *α*7, its persistence did not differ significantly from that of *α*9. Given the high prevalence and oncogenic potential of *α*9, continued postoperative surveillance remains important. These findings support the potential utility of HPV persistence together with pathological features, particularly lesion extent and surgical margin status, in postoperative risk assessment. Further prospective multicenter studies are needed to confirm these associations and determine their value for individualized postoperative surveillance.

## Data Availability

The raw data supporting the conclusions of this article will be made available by the authors, without undue reservation.
