# Supplementary material for: Risk factors for HR-HPV persistence and residual or recurrent high-grade squamous intraepithelial neoplasia after LEEP
Source: Front Med (Lausanne). 2026 Sep 24;13:1897304. doi: 10.3389/fmed.2026.1897304 (PMC13647321; doi:10.3389/fmed.2026.1897304)
Supplement: Supplementary file 1 [file Table1.docx]

**Supplementary Material**

**Risk Factors for Type-Specific HR-HPV Persistence and Residual or Recurrent High-grade Squamous Intraepithelial Neoplasia After LEEP**

**Liya Wang****^1,2,3,#,^*, Yiran Zhang^1,2,3,#^, Yi Chen^1,2,3^, Ying Xu^1,2,3^, Lei Bao^1,2,3^, Dan Cao^1,2,3,^*, Dan Wu^1,2,3,^***

^1^The Center for Cervical Disease, The International Peace Maternity and Child Health Hospital, School of Medicine, Shanghai Jiao Tong University, Shanghai 200030, China

^2^Shanghai Key Laboratory of Embryo Original Disease, Shanghai 200030, China

^3^National Key Clinical Specialty Project (2026) Construction of Innovative Diagnosis and Treatment Technology for Gynecologic Oncology and Standardized System for Oncofertility

***Correspondence:**

Liya Wang, [annya1985010@sina.com](mailto:annya1985010@sina.com)

Dan Cao, [caodan1272@163.com](mailto:caodan1272@163.com)

Dan Wu, [drwudan2026@163.com](mailto:drwudan2026@163.com)

^#^These authors have contributed equally to this work and share first authorship.

[**Supplementary**](https://pmc.ncbi.nlm.nih.gov/articles/instance/11562306/bin/12951_2024_2986_MOESM1_ESM.docx) **table 1. HPV species-specific rates of persistence and multiple infection.**

| **HPV Types** | **HPV genotypes before LEEP (%)** | **Multiple hrHPV infection before LEEP (%)** | **Persistence of the same type after LEEP (%)** |
| --- | --- | --- | --- |
| Species α9 |  |  |  |
| HPV16 | 373 (41.3) | 134 (35.9) | 55/373 (14.7) |
| HPV52 | 247 (27.4) | 115 (46.6) | 53/247 (21.5) |
| HPV58 | 159 (17.6) | 70 (44.0) | 46/159 (28.9) |
| HPV33 | 89 (9.9) | 49 (55.1) | 14/89 (15.7) |
| HPV31 | 61 (6.8) | 34 (55.7) | 11/61 (18.0) |
| HPV35 | 25 (2.8) | 19 (76.0) | 7/25 (28.0) |
| Species α7 |  |  |  |
| HPV18 | 68 (7.5) | 45 (66.2) | 4/68 (5.9) |
| HPV45 | 7 (0.8) | 5 (71.4) | 1/7 (14.3) |
| HPV39 | 35 (3.9) | 27 (77.1) | 11/35 (31.4) |
| HPV59 | 19 (2.1) | 14 (73.7) | 6/19 (31.6) |
| HPV68 | 39 (4.3) | 29 (74.4) | 7/39 (17.9) |
| Species α5/6 |  |  |  |
| HPV51 | 46 (5.1) | 33 (71.7) | 17/46 (37.0) |
| HPV53 | 55 (6.1) | 45 (81.8) | 20/55 (36.4) |
| HPV56 | 34 (3.8) | 25 (73.5) | 10/34 (29.4) |
| HPV66 | 31 (3.4) | 21 (67.7) | 8/31 (25.8) |

**Supplementary table 2. GEE-adjusted comparison of multiple infection rates by HPV species (Bonferroni-adjusted pairwise comparisons).**

| **Comparison** | **OR (95% CI)** | **Adjusted *P* value** |
| --- | --- | --- |
| α5/6 vs. α7 | 1.18 (0.65-2.14) | 1 |
| α5/6 vs. α9 | 3.74 (2.38-5.86) | <0.001 |
| α7 vs. α9 | 3.17 (2.06-4.86) | <0.001 |

**Supplementary table 3. GEE-adjusted comparison of species-specific HR-HPV persistence by HPV species (Bonferroni-adjusted pairwise comparisons).**

| **Comparison** | **OR (95% CI)** | **Adjusted *P* value** |
| --- | --- | --- |
| α5/6 vs. α7 | 2.30 (1.09-4.85) | 0.024 |
| α5/6 vs. α9 | 1.61 (0.95-2.71) | 0.091 |
| α7 vs. α9 | 0.70 (0.40-1.24) | 0.403 |

**Supplementary table 4. Risk factors associated with residual or recurrent HSIL after LEEP.**

| **Characteristics** |  | **without recurrence/residual (n=871)** | **with recurrence/residual (n=32)** | ***P*-value** |
| --- | --- | --- | --- | --- |
| Age (years) |  | 40.0 [34.5, 49.0] | 52.0 [42.0, 59.0] | <0.001 |
| Menopausal | Postmenopausal | 198 (92.1%) | 17 (7.9%) | <0.001 |
|  | Premenopausal | 673 (97.8%) | 15 (2.2%) |  |
| Number of pregnancies | ＜3 | 533 (97.6%) | 13 (2.4%) | 0.031 |
|  | ≥3 | 338 (94.7%) | 19 (5.3%) |  |
| Parity | ＜2 | 655 (97.2%) | 19 (2.8%) | 0.07 |
|  | ≥2 | 216 (94.3%) | 13 (5.7%) |  |
| Preoperative HPV type | Single | 602 (96.6%) | 21 (3.4%) | 0.822 |
|  | Multiple | 269 (96.1%) | 11 (3.9%) |  |
| Persistent HPV infection | Yes | 182 (86.7%) | 28 (13.3%) | <0.001 |
|  | No | 689 (99.4%) | 4 (0.6%) |  |
| Cervical cytology | NILM | 339 (97.7%) | 8 (2.3%) | 0.04 |
|  | ASCUS/AGC | 208 (97.7%) | 5 (2.3%) |  |
|  | LSIL | 143 (96.6%) | 5 (3.4%) |  |
|  | ASC-H | 81 (93.1%) | 6 (6.9%) |  |
|  | HSIL | 100 (92.6%) | 8 (7.4%) |  |
| Cervical transformation zone (CTZ) | I | 361 (97.8%) | 8 (2.2%) | 0.157 |
|  | II | 236 (95.9%) | 10 (4.1%) |  |
|  | III | 274 (95.1%) | 14 (4.9%) |  |
| Number of cervical quadrants involved | 0 | 116 (97.5%) | 3 (2.5%) | 0.011 |
|  | 1 | 562 (97.4%) | 15 (2.6%) |  |
|  | 2 | 134 (92.4%) | 11 (7.6%) |  |
|  | 3 | 30 (100.0%) | 0 (0.0%) |  |
|  | 4 | 29 (90.6%) | 3 (9.4%) |  |
| Endocervical involvement | Negative | 548 (98.2%) | 10 (1.8%) | <0.001 |
|  | Positive (CIN2-3) | 215 (92.3%) | 18 (7.7%) |  |
|  | Positive (CIN1) | 108 (96.4%) | 4 (3.6%) |  |
| Glandular involvement | Yes | 525 (95.6%) | 24 (4.4%) | 0.136 |
|  | No | 346 (97.7%) | 8 (2.3%) |  |
| Colposcopic pathology | Chronic cervicitis | 12 (92.3%) | 1 (7.7%) | 0.12 |
|  | CIN1 | 127 (98.4%) | 2 (1.6%) |  |
|  | CIN2 | 427 (97.3%) | 12 (2.7%) |  |
|  | CIN3 | 305 (94.7%) | 17 (5.3%) |  |
| Postoperative pathology | CIN2 | 454 (97.8%) | 10 (2.2%) | 0.058 |
|  | CIN3 | 413 (94.9%) | 22 (5.1%) |  |
|  | CIS | 4 (100.0%) | 0 (0.0%) |  |
| Surgical margin | Negative | 755 (98.7%) | 10 (1.3%) | <0.001 |
|  | Ectocervical margin | 39 (90.7%) | 4 (9.3%) |  |
|  | Endocervical margin | 64 (83.1%) | 13 (16.9%) |  |
|  | Basal margin | 13 (72.2%) | 5 (27.8%) |  |

**Supplementary table 5. Distribution of preoperative HPV genotype with residual or recurrent HSIL**

| **α group** | **HPV types** | **Number of infections** | **Total number** |
| --- | --- | --- | --- |
| Species α9 | 16 | 16 | 42 (73.7%) |
|  | 33 | 5 |  |
|  | 35 | 4 |  |
|  | 52 | 9 |  |
|  | 58 | 8 |  |
|  | 31 | 0 |  |
| Species α5/6 | 51 | 4 | 10 (17.5%) |
|  | 53 | 4 |  |
|  | 56 | 2 |  |
|  | 66 | 0 |  |
| Species α7 | 18 | 1 | 5 (8.8%) |
|  | 45 | 0 |  |
|  | 39 | 1 |  |
|  | 59 | 0 |  |
|  | 68 | 3 |  |
